# Myocardial Injury Promotes Matrix Metalloproteinase-9 Activity in the Renal Cortex in Preclinical Models of Acute Myocardial Infarction

**DOI:** 10.1007/s12265-021-10114-y

**Published:** 2021-03-29

**Authors:** Xiaoying Qiao, Shreyas Bhave, Lija Swain, Elric Zweck, Lara Reyelt, Paige Crowley, Shiva K. Annamalai, Aditya Chennjorwala, Michele Esposito, Allen Razavi, Sina Foroutanjazi, Cody Machen, Katherine Thayer, Lena Jorde, Richard H. Karas, Navin K. Kapur

**Affiliations:** Molecular Cardiology Research Institute, Surgical and Interventional Research Laboratories, and The CardioVascular Center, Tufts Medical Center, 800 Washington Street Box # 80, Boston, MA 02111 USA

**Keywords:** Myocardial infarction, Cardio-renal, Kidney injury, Hemodynamics, Mechanical support

## Abstract

New mechanistic insight into how the kidney responds to cardiac injury during acute myocardial infarction (AMI) is required. We hypothesized that AMI promotes inflammation and matrix metalloproteinase-9 (MMP9) activity in the kidney and studied the effect of initiating an Impella CP or veno-arterial extracorporeal membrane oxygenation (VA-ECMO) before coronary reperfusion during AMI. Adult male swine were subjected to coronary occlusion and either reperfusion (ischemia-reperfusion; IR) or support with either Impella or VA-ECMO before reperfusion. IR and ECMO increased while Impella reduced levels of MMP-9 in the myocardial infarct zone, circulation, and renal cortex. Compared to IR, Impella reduced myocardial infarct size and urinary KIM-1 levels, but VA-ECMO did not. IR and VA-ECMO increased pro-fibrogenic signaling via transforming growth factor-beta and endoglin in the renal cortex, but Impella did not. These findings identify that AMI increases inflammatory activity in the kidney, which may be attenuated by Impella support.

## Introduction

Acute kidney injury (AKI) after acute myocardial infarction (AMI) is associated with an increased risk of both short- and long-term mortality [1–3]. Proposed mechanisms for AKI in AMI include hypotension, contrast-associated nephropathy, and sympathetic nervous activation; however, recent data suggests that AKI may develop independently of these factors [4, 5]. New mechanistic insight into how the kidney responds to cardiac injury during AMI is required.

Over the past decade, use of trans-valvular pumps and veno-arterial extracorporeal membrane oxygenation (VA-ECMO) for patients with AMI has grown exponentially [6–8]. Trans-valvular pumps (Impella; Abiomed, Danvers, MA) transfer rotational kinetic energy to blood and generate flow from the left ventricle (LV) into the ascending aorta. VA-ECMO drains blood from the venous system and returns oxygenated blood into the descending aorta. Both devices increase systemic blood pressure and end-organ perfusion in AMI. We recently reported that Impella, but not VA-ECMO, reduces myocardial infarct size by reducing cardiac workload, increasing myocardial perfusion and activating a cardio-protective signaling program in preclinical models of AMI [9–11]. Recent reports have suggested a potential reno-protective effect of Impella support during high-risk coronary intervention [12, 13]; however, the mechanisms underlying this protective effect remain poorly understood.

We and others have shown that coronary reperfusion rapidly increases myocardial and circulating levels of matrix metalloproteinase-9 (MMP-9) in AMI [10, 14, 15]. We have also reported that activating an Impella before reperfusion reduces LV workload, known as LV unloading, infarct size, and myocardial MMP-9 activity in AMI, but VA-ECMO does not [10, 16]. Other studies have suggested that elevated MMP levels and activation of the transforming growth factor beta-1 (TGFβ-1) system promote maladaptive cardiac remodeling and contribute to kidney injury [17–21]. However, the role of MMP-9 and TGFβ-1 in AKI during the acute phase of myocardial injury remains poorly understood. While it is known that renal ischemia induces MMPs and plays a critical role in kidney injury, whether acute myocardial ischemia can activate MMPs in the kidneys is unknown [22]. Kidney injury molecule 1 (KIM-1) is a well-established marker of kidney damage [23, 24]. Circulating and urinary levels of KIM-1 are indicative of renal damage [25, 26].

With this background in mind, we hypothesized that acute myocardial ischemia and reperfusion injury increase MMP-9 and TGFβ-1 activity in the kidney and further that reducing myocardial injury with LV unloading the left ventricle before reperfusion reduces MMP activity and profibrotic signaling in the kidney during AMI.

## Methods

### Experimental Protocol of Myocardial Infarction and Mechanical Circulatory Support

Studies were conducted in adult, male Yorkshire swine. The study protocol was approved by the Institutional Animal Care and Use Committee (IACUC) at Tufts Medical Center. All experiments were performed according to the committee’s guidelines. Animals were pre-medicated with Telazol (0.8 ml/kg, intramuscular). General anesthesia was induced and maintained with isoflurane (1–2%). All animals were intubated and mechanically ventilated (Harvard Apparatus Inc.) with room air and supplemented oxygen to maintain physiologic pH and oxygen saturation. Surface electrocardiography leads, an orogastric tube, peripheral 18 G venous catheters, and a rectal thermistor were placed in all animals. A Foley catheter with a side port for urine sampling was surgically implanted through a small incision into the bladder. Heating pads were used as needed to maintain a core body temperature > 99° F. Vascular access sheaths were then deployed into the right internal jugular vein (10Fr), left carotid artery (7Fr), and both femoral arteries (7Fr) and veins (10Fr). Unfractionated heparin boluses with a goal activated clotting time of 300–400 s, continuous lidocaine infusion (1 mg/kg), and noradrenaline (0.16 mcg/min) were initiated in all animals. A 6Fr Judkins right coronary catheter (Boston Scientific) engaged the left coronary artery via the right femoral artery, and baseline angiograms were recorded. A pressure wire was delivered into the distal left anterior descending artery (LAD), and a 3.0 × 8 mm angioplasty balloon (Boston Scientific) was deployed in the mid-LAD after the first diagonal branch with angiographic confirmation of LAD occlusion. Coronary angiography performed immediately after reperfusion and again after the end of the study protocol confirmed patency of the LAD. Following reperfusion, the LAD balloon was left in position for repeat balloon occlusion during Evans blue counterstaining.

**Fig. 1:**
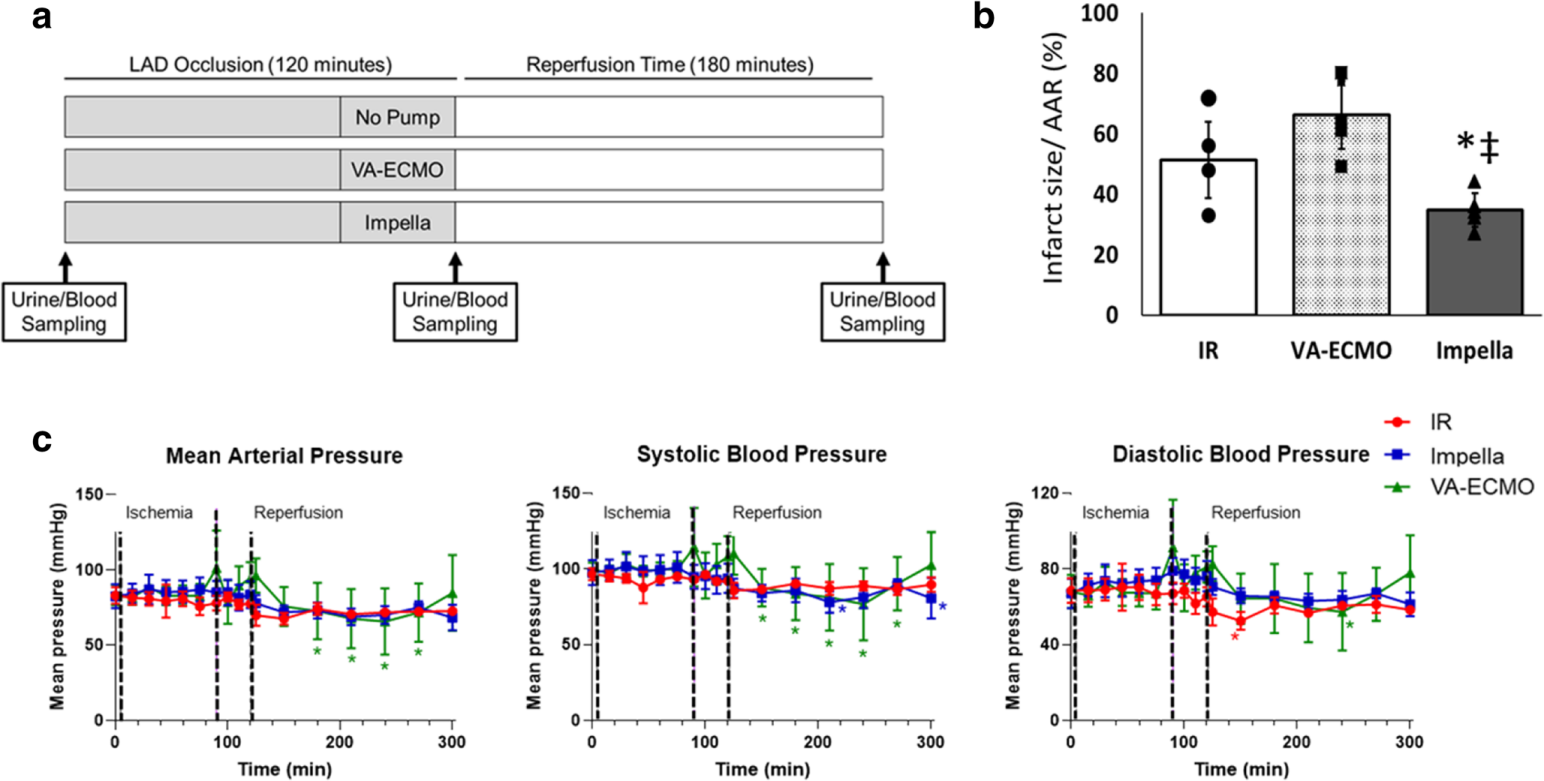
Infarct size associated with Impella or VA-ECMO plus delayed reperfusion. (**a**) Study design. (**b**) Infarct size normalized to the area at risk for the ischemia-reperfusion (IR), Impella, and VA-ECMO groups (*n* = 5/group; *p = 0.02, IR alone vs Impella; † *p* = 0.01, VA-ECMO vs Impella). (**c**) Line graphs illustrating changes in mean arterial pressure and aortic systolic and diastolic pressures (*n* = 5/group; *p < 0.05; **p < 0.01 vs. Baseline hemodynamic values are at time point zero

To explore the effects of mechanical support with Impella or VA-ECMO prior to reperfusion in AMI, 15 swine underwent 120 min of LAD occlusion followed by 180 min of reperfusion. Following 90 min of LAD occlusion, subjects were randomly assigned to have continued occlusion alone for 30 min (ischemia-reperfusion; IR), continued occlusion for 30 min with activation of an Impella CP, or continued occlusion for 30 min with activation of VA-ECMO (*n* = 5/group; Fig. 1a). In the two device arms, pumps remained active throughout the 180 min after reperfusion. The Impella CP was inserted via a 14 French (Fr) sheath in the right carotid artery and activated at maximal support (44,000 rotations per minute (RPM) or P-level 8). VA-ECMO was initiated using a 19 Fr arterial cannula and 21 Fr multi-stage venous cannula in the left femoral artery and right femoral vein, respectively. VA-ECMO was activated at 7500 RPM using an extracorporeal centrifugal flow pump (CardiacAssist Inc., Pittsburgh, PA) and a membrane oxygenator (Quadrox, Maquet Inc., Mahwah, NJ).

### Myocardial Infarct Quantification

After 180 min of reperfusion, the LAD was balloon occluded, followed by intra-coronary delivery of Evans blue. Animals were then euthanized, and myocardial infarct size was quantified using digital photography across LV cross-sectional slices incubated in 1% triphenyltetrazolium chloride (TTC). Three blinded reviewers (XQ, LS, ME) quantified the total myocardial area, area-at-risk, and infarct zone using digital planimetry to identify the infarct percentage relative to the area at risk.

### Left Ventricular Hemodynamic Assessment

LV pressure and volume were recorded throughout the study protocol. For LV pressure and volume measurements, a 5Fr-conductance catheter system (Sigma-M; CD Leycom, The Netherlands) deployed via the left carotid was used as we have previously described [27, 28]. Absolute LV volumes were measured by subtracting parallel conductance from total conductance volumes. Stroke volume is calculated as the difference in conductance volumes at +dP/dtmax and –dP/dtmin. LV stroke work was calculated as the product of LV peak systolic pressure and stroke volume. Systemic blood pressure was monitored using fluid-filled manometers in the aortic root and femoral artery.

### Protein Extraction and Immunoblot Analysis

Swine heart and kidney tissues were harvested after euthanasia. LV tissue samples were collected from infarct and non-infarct zones for homogenate preparation. For renal cortex and medulla, kidneys were dissected to separate renal cortex tissue and medullary tissue. For Western blot analysis, proteins were extracted with T-PER Tissue Protein Extraction Reagent (Thermo Scientific). Protein concentrations were determined using the Pierce BCA Protein Assay kit. Proteins were separated via SDS-PAGE 4–15% gel (Bio-Rad; mini-protean TGX Gels, 456-1086) and transferred onto PVDF membrane. The proteins of interest were detected using the following primary and secondary antibodies: pSmad3 (Cell Signaling, 9520 S), Smad2/3 (Cell signaling, 8685S), endoglin (Novus Biologicals, NBP2–22122), GAPDH (Millipore, MAB374), and α tubulin (Cell Signaling, 2144S). Expression of pSmad-3 protein levels were normalized to both total protein levels and GAPDH. HRP-linked secondary antibodies, anti-rabbit IgG (Cell Signaling, 7074S), anti-mouse IgG (Cell signaling 7076S), and anti-goat IgG (R&D Systems, HAF109) were used.

### Quantification of Renal Injury Biomarkers

Serum, urine, plasma, and tissue levels for KIM-1 (MBS2506418, MyBiosource) were quantified through enzyme-linked immunosorbent assay (ELISA) as per the manufacturer’s instruction. KIM-1 in the plasma and urine was quantified using ELISA as per manufacturer’s instruction (MBS2506418, MyBiosource).

### Quantification of MMP-2 and MMP-9 Levels and Activity

MMP-2 and MMP-9 activities in homogenates of swine tissue were determined by zymography. Briefly, gelatin zymography was performed with SDS-PAGE gels containing 1 mg/ml porcine gelatin. Samples from hearts and kidneys were prepared under non-reducing conditions. Gel electrophoresis was performed at 150 V for 1 h. After electrophoresis, the gel was washed in 2.5% Triton X-100 solution with gentle agitation for 6 h at room temperature, followed by incubation in a developing buffer containing 50 mM Tris-HCl (pH 7.5), 0.2 M NaCl, 5 mM CaCl_2_, and 0.2% Brij-35). The gel was agitated at room temperature for 30 min, placed into a fresh developing buffer, and incubated at 37 °C overnight. The following morning, gels were stained with 0.5% Coomassie Brilliant Blue R-250 in 40% methanol and 10% acetic acid for 2–4 h and de-stained in 40% methanol and 10% acetic acid at room temperature. Gelatinolytic bands were quantified by scanning densitometry with NIH ImageJ software. For measuring circulating levels of plasma MMP-2 and 9, blood samples were collected at baseline, occlusion, and after reperfusion (Fig. 1a). Samples were analyzed to determine the circulating levels of MMPs using ELISA (MyBiosource MBS2701188 for MMP-2 and MBS4501118 for MMP-9) as per the manufacturer’s protocol.

### Quantification of Tissue and Circulating Levels of Inflammatory Markers

Plasma and tissue levels of circulating and tissue cytokines IL-6 (MyBiosource, MBS9360517), TNF-α (MyBiosource, MBS043098), IL-1 (MyBiosource, MBS762788), IL-1α (MyBiosource, MBS777753), and INF-γ (MyBiosource, MBS2700897) were quantified through ELISA as per manufacturer’s instruction.

### Statistical Analysis

Data in figures are presented as mean ± SD. Data in tables are displayed as mean ± SD. One-way ANOVA with protected Fisher’s LSD test was used to compare continuous variables between groups. All data within groups over time were analyzed by non-parametric two-way repeated measurement ANOVA or a mixed effects analysis if one or more values were missing at at least one time point. In those graphs, post hoc, data points were compared with baseline using Dunnett’s test or compared between groups using Tukey’s test. Spearman rank correlation was used to calculate correlation coefficients. For the graphs analyzing different concentrations of Kim-1 treatment, two-way ANOVA with Dunnett’s post hoc test was used to compare all concentrations with no treatment. All statistical analyses were carried out in GraphPad Prism Version 8.2.1 (GraphPad Software Inc., La Jolla, CA, USA) or IBM SPSS Statistics Version 25 (International Business Machines Corporation, Armonk, NY, USA). A significance level of *p* < 0.05 was considered significant.

## Results

### Trans-valvular Pumps Reduce Infarct Size in AMI

Compared to myocardial ischemia-reperfusion (IR) or VA-ECMO support for 30 min before reperfusion, Impella CP device activation before reperfusion reduced myocardial infarct size (51 ± 14% vs 62 ± 15% vs 33 ± 7%, IR, VA-ECMO, Impella, respectively, *p* < 0.05 for Impella vs either group; Fig. 1b).

### Hemodynamic Effects of Impella and VA-ECMO in AMI

During LAD occlusion (Fig. 1c), mean arterial pressure and both aortic systolic and diastolic pressures were unchanged compared to baseline values. After reperfusion, mean arterial pressure and aortic systolic pressure were transiently reduced but normalized back to baseline values after 180 min with VA-ECMO support. Compared to baseline values, no change in mean arterial pressure or aortic systolic or diastolic pressures was observed in the IR or Impella groups (Fig. 1c; Table 1). These data suggest that systemic arterial pressure remained largely unchanged during myocardial ischemia and reperfusion injury with or without mechanical circulatory support.

**Table 1:** Hemodynamic data

	Heart rate (bpm)	Ao MAP (mmHg)	LVEDP (mmHg)	LVSW (mmhg)	Device flow (L/min)
Ischemia-reperfusion (IR; *n* = 5)					
Baseline	75 ± 8	84 ± 9	9 ± 3	2891 ± 1020	N/A
90-min occlusion	77 ± 11	81 ± 16	12 ± 6	2812 ± 832	N/A
120 min	73 ± 9^*ˠ*^	82 ± 81	13 ± 5	2950 ± 896	N/A
End of reperfusion	83 ± 9	77 ± 78	13 ± 4	2950 ± 1092	N/A
Impella (*n* = 5)					
Baseline	76 ± 16	83 ± 9	6 ± 4	2831 ± 739	N/A
90-min occlusion (pre-pump)	74 ± 14	87 ± 16	8 ± 1	3014 ± 1005	N/A
120-min occlusion (on pump)	75 ± 10	84 ± 18	4 ± 3#	2245 ± 1428# †	3.1 ± 0.1
End of reperfusion	88 ± 10	68 ± 15	5 ± 2*	1668 ± 988	3.1 ± 0.1
VA-ECMO (*n* = 5)					
Baseline	77 ± 19	92 ± 12	9 ± 8	2992 ± 783	N/A
90-min occlusion (pre-pump)	67 ± 12	80 ± 11	11 ± 5	2309 ± 760	N/A
120-min occlusion (on pump)	72 ± 28	86 ± 17	10 ± 7	2422 ± 335	5.1 ± 0.8
End of reperfusion	82 ± 7	93 ± 20	2 ± 1*	1631 ± 789	4.9 ± 1.1

Heart rate (bpm; beats per minute); aortic (Ao) mean arterial pressure (MAP); left ventricular end-diastolic pressure (LVEDP); left ventricular stroke work (LVSW); device flow (liters per minute; L/min). *, *p* < 0.05 vs IR; #, 90 min vs 120 min; ‡, *p* < 0.05 vs baseline; †, *p* < 0.05 vs VA-ECMO

IR did not reduce LV stroke work (LVSW: 2812 ± 832 vs 2950 ± 896 mmHg-mL, *p* = 0.14) or LV end-diastolic pressure (LVEDP: 12 ± 6 vs 13 ± 5 mmHg, *p* = 0.21) between 90 and 120 min of LAD occlusion (Table 1). Impella activation generated estimated flows of 3.1 ± 0.1 L/min and when compared to pre-activation levels significantly reduced LVSW (3014 ± 1005 vs 2245 ± 1428 mmHg-mL, *p* = 0.04) and LVEDP (8 ± 1 vs 4 ± 3 mmHg, *p* = 0.01) between 90 and 120 min of LAD occlusion with the pump activated. VA-ECMO generated flows of 5.1 ± 0.8 LPM without significantly reducing LVSW (2202 ± 689 vs 2422 ± 335 mmHg-mL, *p* = 0.52) or LVEDP (11 ± 5 vs 10 ± 7 mmHg, *p* = 0.28) between 90 and 120 min of LAD occlusion with the pump activated.

### Renal Cortex Mirrors Cardiac Inflammatory Markers Post AMI

**Fig. 2:**
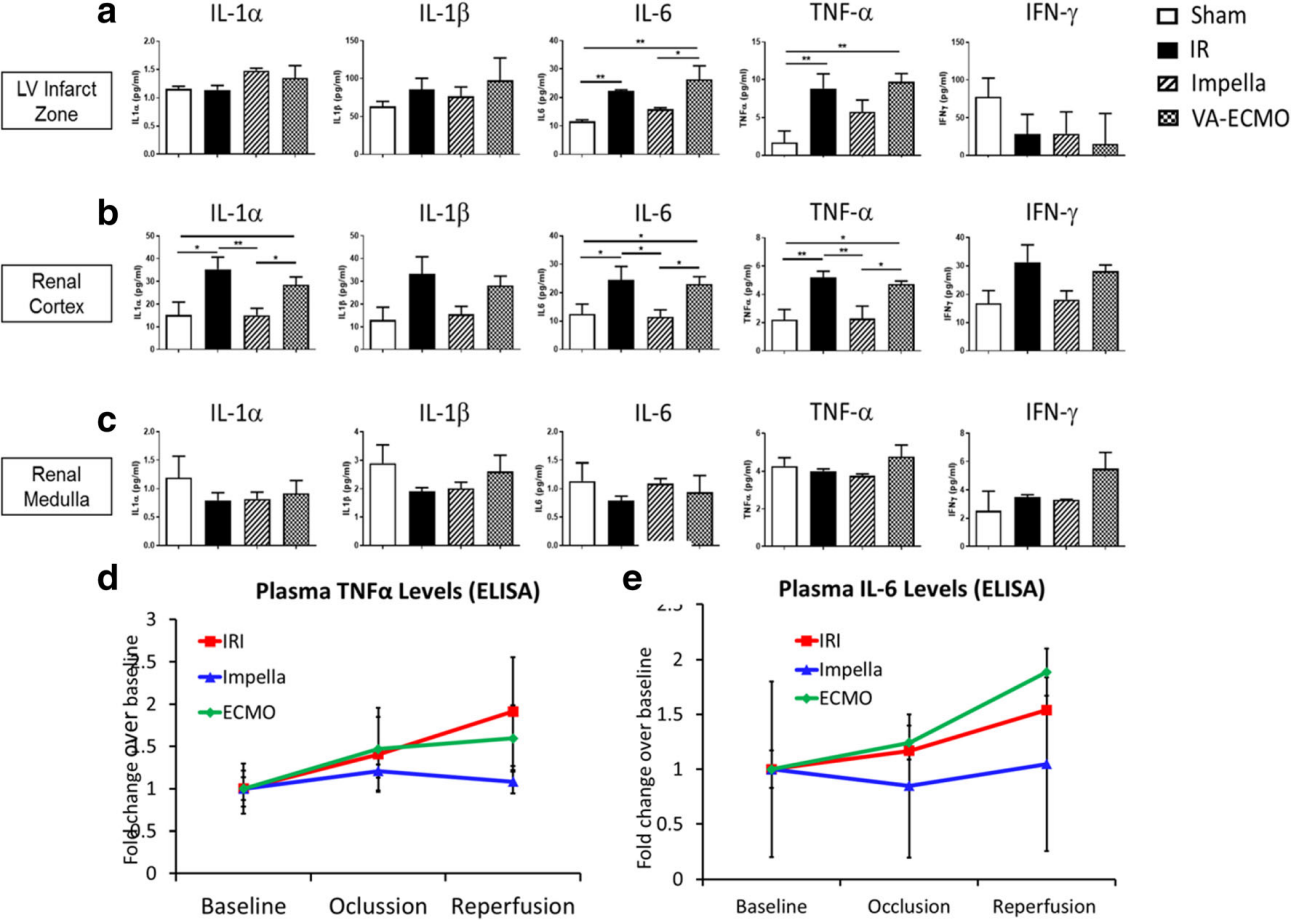
Levels of inflammatory markers. Protein levels of interleukin (IL)-1α, IL-1β, IL-6, tumor necrosis factor-α (TNF-α), and interferon gamma (IFN-γ) quantified by ELISA in the (**a**) LV infarct zone, (**b**) renal cortex, and (**c**) renal medulla. Circulating plasma levels of (**d**) TNFα and (**e**) IL-6 measured by ELISA. *p < 0.05, **p < 0.01, ***p < 0.001. One-way ANOVA with protected Fisher‘s LSD

To determine whether a broader shift in inflammation in the heart is reflected in the kidney during AMI, we quantified levels of inflammatory markers and observed increased IL-6 and TNF-α levels in the heart and renal cortex in the IR and VA-ECMO groups, but not in the Impella group (Fig. 2). Circulating plasma levels of TNF-α and IL-6 remained unchanged.

### AMI Increases MMP-9 Activity in Both the Myocardial Infarct Zone and Renal Cortex

**Fig. 3:**
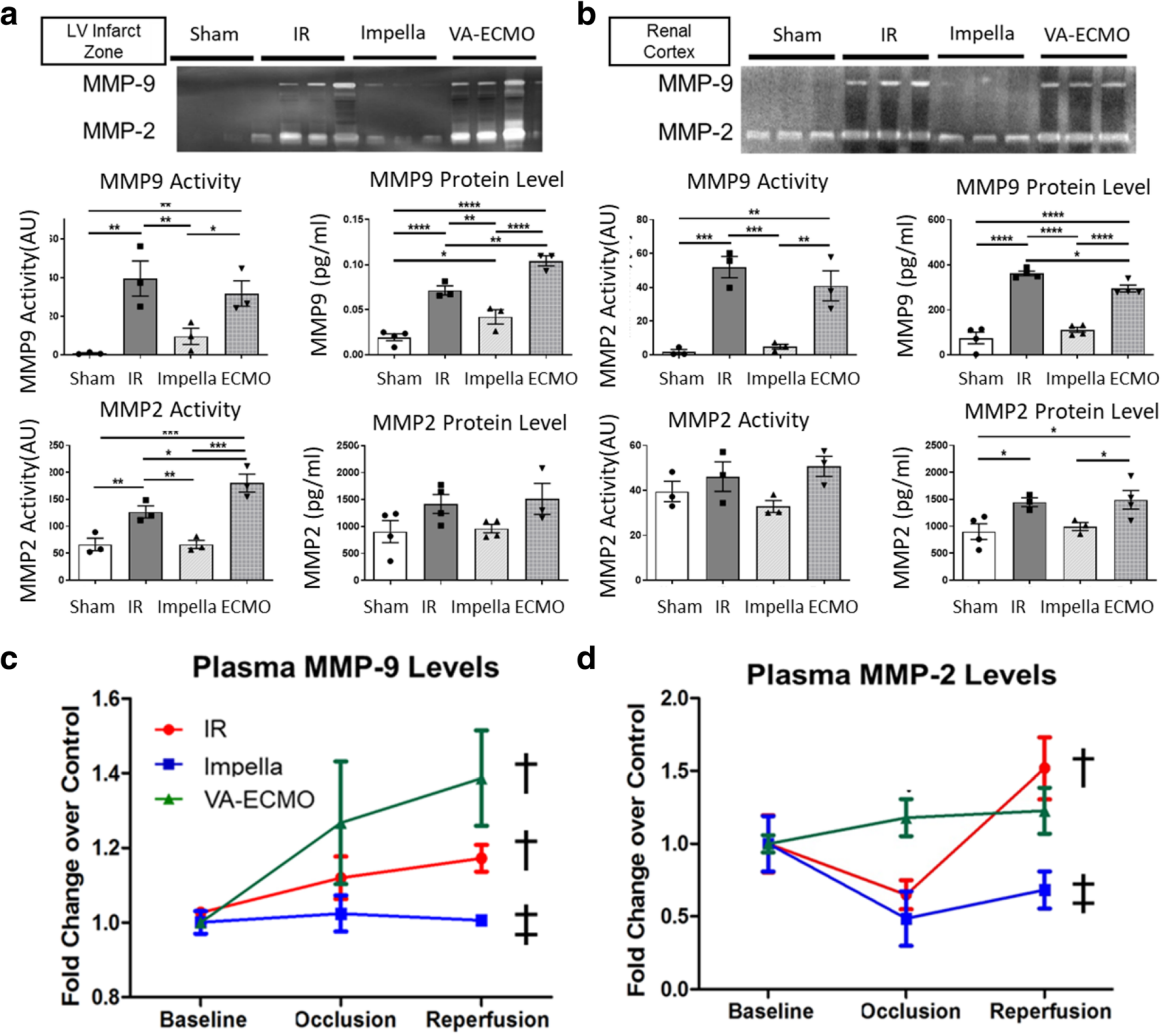
MMP activity in the renal cortex. (**a**) Zymography gel images illustrate matrix metalloproteinase (MMP)-9 and MMP-2 activity in the myocardial infarct zone. Graphs quantify MMP-9 and MMP-2 activity levels and total protein levels by ELISA from the myocardial infarct zone. (**b**) Zymography gel images illustrate MMP-9 and MMP-2 activity in the renal cortex. Graphs quantify MMP-9 and MMP-2 activity and total protein levels by ELISA from the renal cortex. (**c**–**d**) Circulating levels of MMP-9 and MMP-2 are shown as fold change from baseline after 120 min of left anterior descending artery occlusion and 180 min of coronary reperfusion *p < 0.05, **p < 0.01, ***p < 0.001, ****p < 0.0001. One-way ANOVA with protected Fisher‘s LSD; † < 0.05 versus baseline; ‡ <0.05 IR versus Impella

Next, we quantified the expression and activity levels of MMP-2 and MMP-9 in the heart and kidney. Compared to sham controls, IR and VA-ECMO support increased both MMP-9 expression and activity in both the LV infarct zone and the renal cortex (Fig. 3a–b). Compared to sham controls, IR and VA-ECMO increased MMP-2 activity in the LV infarct zone, but not the renal cortex. In contrast, compared to IR or VA-ECMO support, Impella support significantly reduced MMP-2 and MMP-9 protein levels and activity in both the LV infarct zone and renal cortex (Fig. 3a–b). Compared to baseline values, IR and VA-ECMO increased circulating MMP-9 levels, at the end of reperfusion, but Impella did not. Compared to baseline values, only IR increased circulating MMP-2 levels (Fig. 3c–d). These data suggest that myocardial IR increases MMP-9 activity in both the heart and kidney and further that LV unloading attenuates MMP-9 activity in both the heart and kidney during AMI.

### AMI Increases Urinary KIM-1 Levels Indicating Renal Damage

**Fig. 4:**
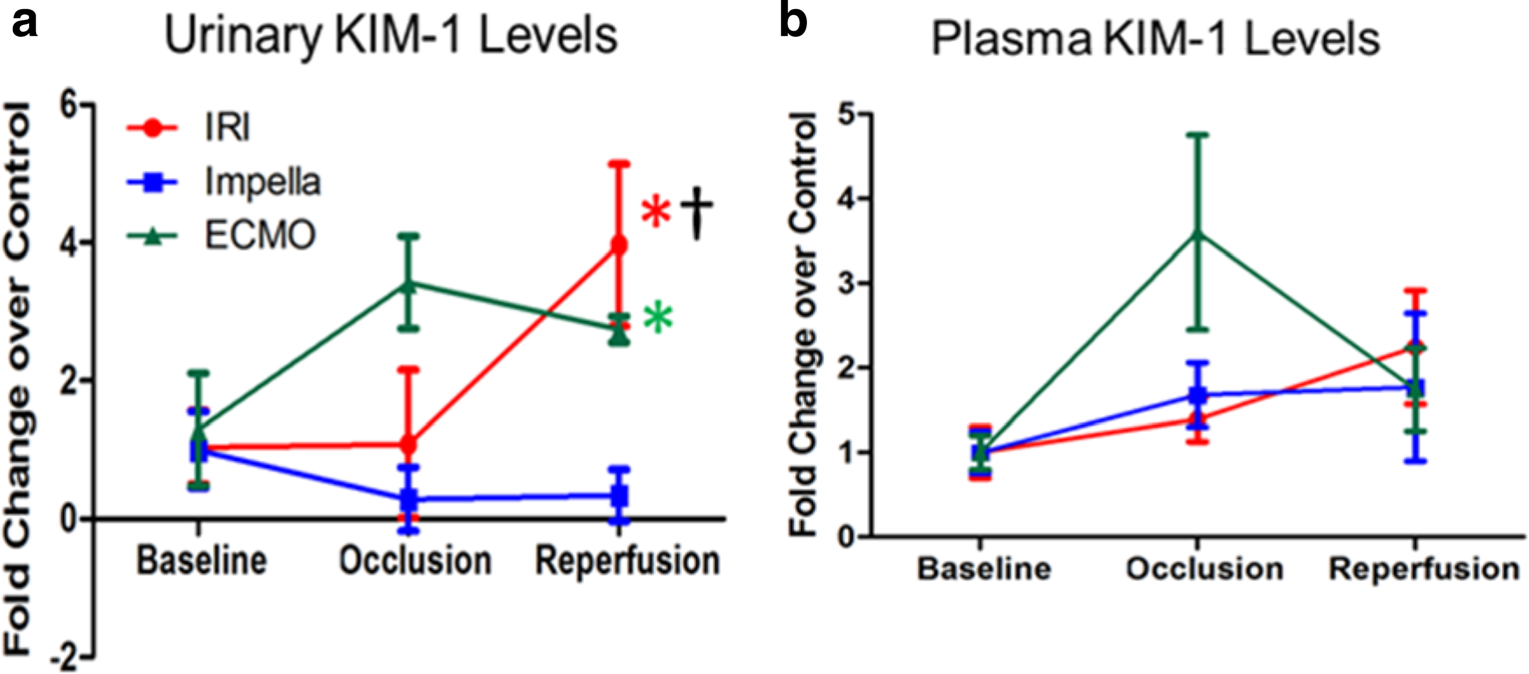
Urinary and circulating levels of KIM-1. (**a**) Urinary and (**b**) plasma levels of kidney injury molecule-1 (KIM-1) are shown as fold change from baseline after 120 min of left anterior descending artery occlusion and 180 min of coronary reperfusion (*n* = 5/group). *p < 0.05 versus baseline; # *p* < 0.05 ECMO versus Impella; † < 0.05 IR vs. Impella

Because urinary KIM-1 levels correlate with renal tubular damage in the setting of chronic heart failure, cardiac surgery, and after myocardial infarction [23, 24, 29–31], we investigated urinary and circulating levels of KIM-1. Compared to baseline values, IR increased urinary levels of KIM-1 (Fig. 4a). Compared to baseline values, VA-ECMO increased urinary KIM-1 levels within 30 min of device activation and at the end of reperfusion. Urinary KIM-1 levels directly associated with infarct size (*R* = 0.67, *r*^2^ = 0.42, *p* = 0.03). Circulating plasma levels of KIM-1 remained unchanged (Fig. 4b).

### AMI Increases Pro-Fibrogenic Signaling in the Renal Cortex

**Fig. 5:**
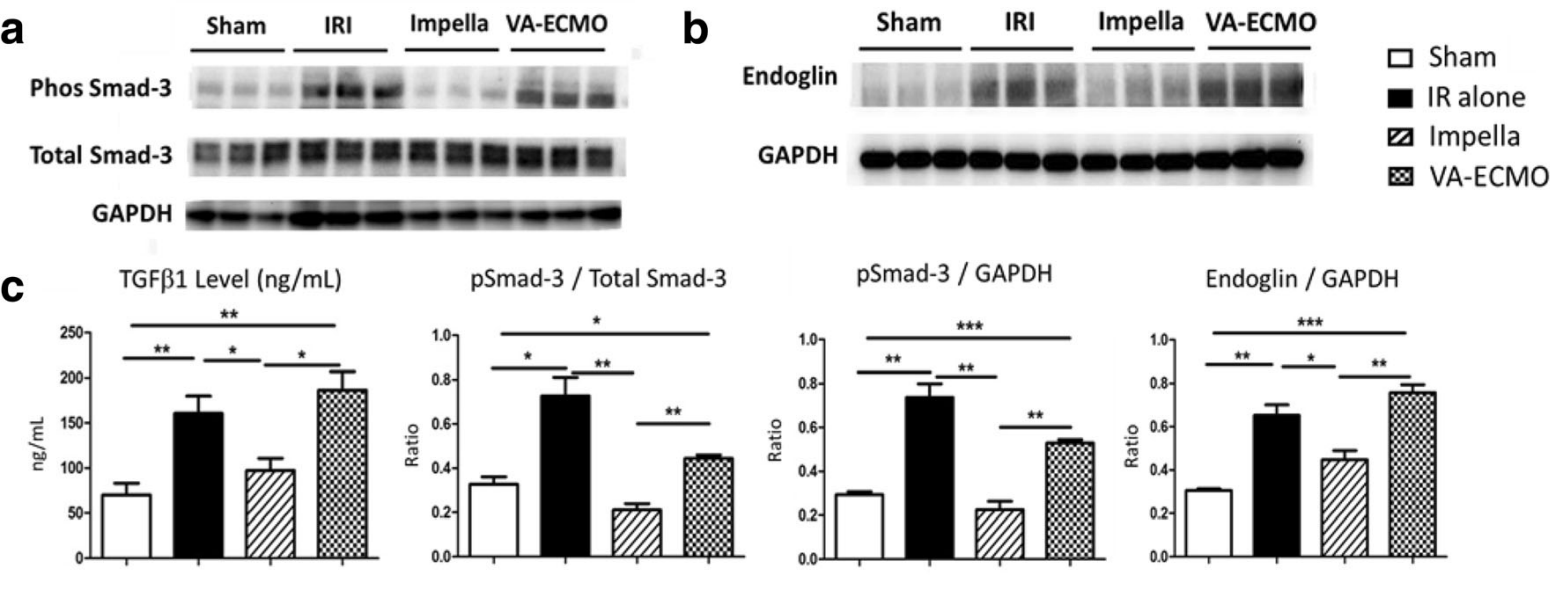
Signaling via TGFβ1, endoglin, and Smad-3. Immunoblots of (**a**) phosphorylated and total Smad-3 and (**b**) endoglin from the renal cortex are shown. (**c**) TGFβ1 protein levels and quantification graphs for phosphorylated Smad-3 and endoglin are shown. *p < 0.05, **p < 0.01, ***p < 0.001, ****p < 0.0001. One-way ANOVA with protected Fisher‘s LSD

Compared to sham controls, IR or VA-ECMO support during AMI increased TGFβ-1 mRNA expression (data not shown) and levels of TGFβ-1 protein, pSmad-3, and endoglin protein levels in the renal cortex (Fig. 5a–c). Compared to IR or VA-ECMO, Impella reduced levels of TGFβ-1, endoglin, and pSmad-3 in the renal cortex.

## Discussion

We introduce new data showing that acute myocardial ischemia and reperfusion injury (IR) increase TNF-a, IL-6, and MMP-9 levels in the LV and renal cortex. Activation of a trans-valvular Impella CP pump, but not VA-ECMO, before reperfusion reduced myocardial infarct size and attenuated an increase in TNF-a and IL-6 levels and MMP-9 levels and activity in the LV and renal cortex. We further observed that urinary KIM-1 levels were increased by myocardial ischemia and reperfusion injury in the presence and absence of VA-ECMO but were unchanged compared to baseline values with Impella CP activation. Urinary KIM-1 levels also correlated directly with myocardial infarct size. We next explored signaling activity in the renal cortex and observed that IR increased levels of TGFβ1, endoglin, and phosphorylated Smad-3, suggesting a pro-fibrogenic signaling environment. Impella CP, but not VA-ECMO, activation attenuated increased levels of these signaling effectors in the renal cortex. These data identify for the first time that expression of inflammatory mediators is shared between the heart and kidney during the acute phase of myocardial ischemia and reperfusion injury and triggers profibrotic signaling in the renal cortex. Future studies are required to test the translational relevance of these observations and to study the impact of short-term circulatory support devices such as Impella or VA-ECMO on kidney injury in AMI.

This is the first report to identify an association between acute myocardial ischemia-reperfusion injury and renal MMP9 activation. Pioneering work by Spinale and colleagues showed that MMP-9 activity is increased within 120 min after the onset of myocardial ischemia in swine and may be related to the influx of inflammatory cells including neutrophils [14]. These findings were further confirmed in patients with acute myocardial infarction where circulating MMP-9 levels were significantly increased within hours after the onset of myocardial ischemia [15]. Our findings now extend this work by showing that MMP-9 activity in the renal cortex mirrors the myocardium and further that reducing myocardial injury with left ventricular unloading attenuates MMP-9 activity in both the heart and kidney. Future studies are needed to better understand the implications of this crosstalk pattern between heart and kidney.

MMPs play an important role in post injury repair mechanism. Many studies have shown the important role they play in recovery post AMI. Interestingly overactive MMPs lead to maladaptive remodeling of the heart and LV failure [15]. Along with their role in AMI, MMPs play a critical role in renal diseases. Renal ischemia increases the MMP activity in the kidney [22]. MMP 9 and 2 degrade ECM and hence allow the remodeling of the kidney. Elevated levels of MMP2 and 9 have been associated with renal fibrosis in CKD [14, 19]. Reports have suggested that activation of ECMO may lead to platelet dysregulation causing increased levels of other MMPs but not MMP-9 [32]. Whether this increase is associated with kidney dysfunction is not known.

Multiple studies have explored the association of KIM-1 levels and kidney injury in the setting of heart failure, myocardial infarction, and after cardiac surgery [4, 5, 23, 29]. However, changes in KIM-1 levels during the acute phase of myocardial ischemia-reperfusion injury remain largely unexplored. Importantly, trans-valvular unloading of the LV reduced myocardial damage and attenuated renal inflammation, MMP9 levels, and activity. Furthermore, it also abrogated pro-fibrogenic signaling in the renal cortex. Notably, VA-ECMO increased urinary KIM-1 levels within 30 min of activation with elevations sustained through 120 min after reperfusion, which may suggest a distinct mechanism involving the extracorporeal bypass circuit used with VA-ECMO on KIM-1 shedding in the setting of AMI. Future studies are required to explore the long-term effects of early MMP-9 and KIM-1 activity in the kidney and LV unloading on kidney biomarkers and renal remodeling after myocardial ischemia and reperfusion injury.

**Fig. 6:**
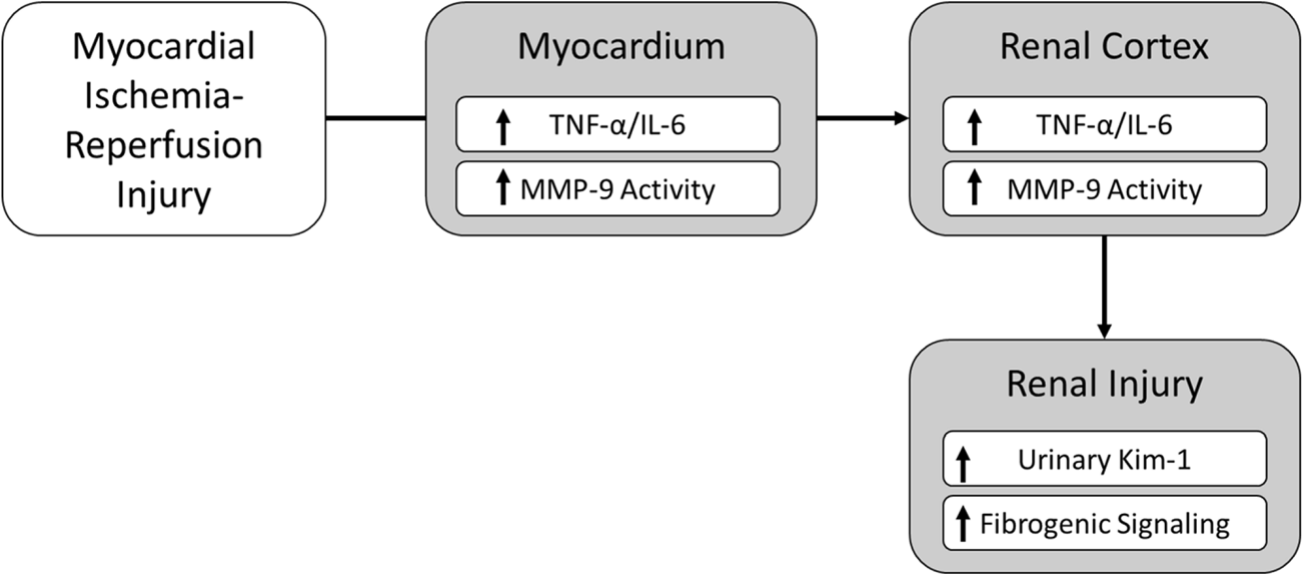
Schematic model of the cardio-renal cross talk and renal injury post AMI. Myocardial ischemia-reperfusion injury elicits an inflammatory response along with increased MMP-9 levels and activity. This inflammatory response and elevated levels and activity of MMP9 are also mirrored in the renal cortex along with profibrotic signaling and elevated levels of KIM-1

Several reports suggest a reno-protective effect of the Impella device during high-risk coronary intervention [12, 13]; however, the mechanism of benefit remains unknown. In contrast, VA-ECMO has been reported to increase acute kidney injury despite increasing systemic blood flow [33, 34]. We have previously reported and confirmed in this study that trans-valvular pumps such as Impella reduce left ventricular stroke work, but VA-ECMO does not. As a result, initiation of Impella, not VA-ECMO, before reperfusion is associated with reduced infarct size and reperfusion injury. Based on our findings, reduced reperfusion injury is associated with decreased MMP-9 activity in the heart and kidney, which may explain the differential effects of these devices on KIM-1 activity. Whether urinary KIM-1 levels correlate with infarct size requires validation in a larger clinical study. Furthermore, VA-ECMO exposes the total circulation to a large foreign surface area (cannulas, tubing, and oxygenator), which may promote inflammation and neutrophil activation and contribute to cardio-renal damage [33]. Collectively our findings indicate that acute myocardial ischemia and reperfusion injury increase expression of inflammatory cytokines and MMP-9 levels in both the myocardium and renal cortex and further are associated with increased urinary KIM-1 levels and activation of fibrotic signaling pathways in the renal cortex (Fig. 6). Future studies exploring the impact of device activation on renal blood flow and auto-regulation are required.

The impact of Impella on myocardial infarct size and post-infarct cardiac remodeling is under active investigation [35]. The STEMI-Door to Unload Pivotal Trial (NCT03947619) will now test whether LV unloading before reperfusion reduces myocardial infarct size compared to coronary reperfusion alone in patients with anterior STEMI without cardiogenic shock [36]. The Euro-Shock study (NCT03813134) will explore the impact of VA-ECMO on clinical outcomes among patients with STEMI and cardiogenic shock [37]. Our findings suggest that the impact of these mechanical support pumps on acute kidney injury should be investigated in these clinical trials.

Limitations of the current study include the lack of information analyzing renal blood flow and late-term kidney injury in this preclinical model; however, we elected to avoid instrumentation of the kidney, thereby preserving organ integrity for analysis. Furthermore, we studied a small number of animals due to a mortality rate of 30% and associated cost. The use of healthy animal models potentially limits the clinical relevance of these observations. Future studies exploring the long-term impact of AMI and circulatory support on renal function and other biomarkers of kidney injury are required.

In conclusion, we introduce for the first time that MMP-9 activity and pro-fibrogenic signaling via TGFβ-1 is increased in both the heart and kidney within minutes after myocardial reperfusion and further that activation of a trans-valvular Impella CP pump, but not VA-ECMO, attenuates MMP-9 and TGFβ-1 activity in the renal cortex during AMI. These findings provide new mechanistic insight into cardio-renal cross talk during AMI and open new avenues of investigation for renal remodeling during the acute and chronic phases after AMI. With further study, these observations have potentially important implications for the management of patients and clinical trials evaluating the impact of mechanical support devices on renal injury in the setting of high-risk coronary intervention, acute myocardial infarction, or cardiogenic shock.

### Clinical Perspectives

AMI remains a major precursor for long-term heart failure. AKI during AMI can lead to long-term damage to the renal system and further exacerbate the cardiac function leading to failure. This report identifies a potentially important role for mechanical circulatory devices in regulating renal injury during AMI. In AMI, clinical intervention mainly focuses on cardiac injury. Our findings underscore the importance of cardio-renal cross talk and renal injury in AMI.
